# Transcriptomic profiling sheds light on the blue-light and red-light response of oyster mushroom (*Pleurotus ostreatus*)

**DOI:** 10.1186/s13568-020-0951-x

**Published:** 2020-01-18

**Authors:** Huan Wang, Xidan Tong, Fenghua Tian, Chuanwen Jia, Changtian Li, Yu Li

**Affiliations:** 1 Department of Agronomy, Jilin Agricultural University, Changchun, 130118 Jilin China; 2 Department of Plant Protection, Jilin Agricultural University, Changchun, 130118 Jilin China; 3 Department of Plant Pathology, Agriculture College, Guizhou University, Guiyang, 550025 People’s Republic of China

**Keywords:** Blue-light, Red-light, Mushroom, Pileus growth, Gene expression, Glycolysis

## Abstract

Blue light is an important environmental factor that induces mushroom primordium differentiation and fruiting body development. Although blue-light treatment has been applied for the production of oyster mushroom (*Pleurotus ostreatus*), the blue-light response mechanisms of *P. ostreatus* still remain unclear. In the present study, we exposed the primordium of *P. ostreatus* to blue-light, red-light, and dark conditions for 7 days. Subsequently, comparative transcriptomics analysis of the stipe, pileus, and gill under the three light conditions was performed to reveal the gene expression response mechanism of *P. ostreatus* to blue light and red light. The results showed that blue light enhanced the growth and development of all the three organs of *P. ostreatus*, especially the pileus. In contrast, red light slightly (non-significantly) inhibited pileus growth. When compared with red-light and dark treatments, blue-light treatment significantly upregulated gene expression involved in glycolysis/gluconeogenesis, the pentose phosphate pathway and the peroxisome in the pileus, but not in the gill or stipe. Most of the glycolysis and pentose phosphate pathway genes were upregulated in the pileus by blue light. When compared with dark treatment, red-light treatment downregulated the expression of many respiration metabolism genes in the pileus. These results revealed that blue light enhanced the activation of glycolysis and the pentose phosphate pathway, whereas red light weakened glycolysis and pentose phosphate pathway activation. The conclusion can be drawn that blue light improved *P. ostreatus* fruiting body (particularly, the pileus) growth rate via enhancement of glycolysis and the pentose phosphate pathway.

## Introduction

Nutrient, temperature, and light conditions are critical environmental factors for primordium differentiation and fruiting body induction in mushrooms (Sakamoto et al. 2018). The influence of light on mushrooms has been well characterized (Fuller et al. 2015; Sakamoto 2018). The effective wavelength for fruiting body induction in mushrooms includes ultraviolet wavelength (280 nm) and blue light (520 nm) (Durand and Furuya 1985; Kitamoto and Gruen 1976; Sakamoto 2018). However, the inductive effect of light on mycelium differentiation and fruiting body growth is still unclear (Sakamoto 2018). It had been widely reported that blue light has strong effects on fruiting body induction (reviewed by Sakamoto 2018) and metabolism in mushrooms (Kojima et al. 2015). Blue-light responses have also been found in other organisms such as plants, ferns, and prokaryotes (Taiz et al. 2015). Among them, the blue-light response mechanism of plants is well characterized. Blue light affects ion uptake of algae (Taiz et al. 2015), and has strong biological effect on growth and development of higher plants, including chloroplast movements, stomatal opening, enhancement of respiration and phototropism, and photomorphogenesis (Taiz et al. 2015). In plants, cryptochromes are blue-light photoreceptors that modulate the suppression of hypocotyl elongation, promotion of cotyledon expansion, membrane depolarization, inhibition of petiole elongation, anthocyanin production, and circadian clock entrainment (Inoue et al. 2010; Lariguet and Dunand 2005; Taiz et al. 2015). Phototropins are also blue-light photoreceptors in plants, which contain serine/threonine protein kinase domain. They optimize photosynthetic efficiency and promote plant growth, particularly under low-light conditions (Inoue et al. 2010; Lariguet and Dunand 2005). Recent studies have identified red-light and blue-light photoreceptors in fungi (Herrera-Estrella and Horwitz 2007; Purschwitz et al. 2006; Sano et al. 2009; Terashima et al. 2005), and have described photoresponse phenomena related to fruiting body formation (Kamada et al. 2010; Terashima et al. 2005), pigmentation (Corrochano and Garre 2010), sporulation (Corrochano and Garre 2010), phototropism (Corrochano and Garre 2010), the circadian cycle (Dunlap and Loros 2006; Sano et al. 2009), and biosynthesis of secondary metabolites (Kojima et al. 2015).

Most recently, some reports had focused on gene expression regulations involved in the blue-light response of mushrooms (Sakamoto et al. 2018; Xie et al. 2018). (Sakamoto et al. 2018) demonstrated that blue light affects the expression of genes involved in simultaneous hyphal knot formation in *Coprinopsis cinerea*. Xie et al. (2018) reported that blue light regulates the expression of genes encoding carbohydrate-active enzymes (CAZymes) during primordium differentiation into fruiting body. Although previous works have provided some insights into the blue-light response of mushrooms, the molecular mechanisms underlying the response remains unclear. Furthermore, as the blue-light response mechanisms of mushrooms vary among different mushroom species (Sakamoto 2018), the functional significance of these mechanisms must be investigated in each case. To the best of our knowledge, there are no studies on the transcriptomics of blue-light and red-light responses of the oyster mushroom, *Pleurotus ostreatus*. In the present study, we exposed the primordium of *P. ostreatus* to blue-light, red-light, and dark conditions for 7 days, and determined the gene expression response of *P. ostreatus* to blue light and red light by conducting comparative transcriptomics analysis of the stipe, pileus, and gill under these three light conditions.

## Methods

### Liquid spawn preparation

Oyster mushroom (*P. ostreatus*) strain zaoqiu615 was selected because it is a stable commercial strain available in Jilin Province, China. The stock culture of strain zaoqiu615 was maintained on potato dextrose agar (PDA) slants at 25 °C for 7 days. Liquid spawn was prepared by culturing the mycelia in a liquid medium at 23.8 °C for 7 days. The liquid medium (neutral pH) consisted of 100 g/L potato, 15 g/L brown sugar, 10 g/L glucose, 45 g/L wheat bran, 2.5 g/L peptone, 2.0 g/L KH_2_PO4, 1 g/L MgSO_4_, 10 mg/L vitamin B1, and 0.3 mL/L glycerol.

### Substrates preparation and light treatment

The liquid spawn was inoculated into sterilized bags (17 cm × 33 cm) containing the growth medium (purchased from the Mushroom base of Jilin Agricultural University, Changchun, China) for radial mycelial growth and fruiting body cultivation. The growth medium (65% moisture content) consisted of corn cob (24%), wood shavings (35%), wheat bran (24%), corn flour (10%), soybean meal (5%), temperament calcium carbonate (1%), and lime (1%). The bags were placed in a mushroom incubator (Hipoint Corporation, Taiwan) at 24 °C, 55% air moisture, and dark condition for 20 days. After 20 days of inoculation, *P. ostreatus* primordium emerged. At this stage, the bags containing newly emerged primordium were subjected to blue-light, red-light, and dark treatments at 24 °C for 7 days. Blue-light and red-light treatments were conducted using an LED blue lighting unit (430–470 nm) and LED red lighting unit (610–640 nm) in the mushroom incubator (MI302, Hipoint Corporation), respectively. The light intensity was about 50 μmol/m^2^/S. After 7 days of light treatment, the stipe, pileus, and gill of each mushroom were collected for RNA extraction. Fresh weights of the stipe and pileus + gill for each treatment were determined, with 10 mushrooms for each treatment.

### RNA sequencing

Each treatment and each tissue comprised three biological replicates, and each biological replicate consisted of three mushrooms. A total of 2 μg of RNA per sample were used as the input material for RNA sample preparations. Sequencing libraries were generated using NEBNext^®^ Ultra™ RNA Library Prep Kit for Illumina^®^ (#E7530L, NEB, USA) following the manufacturer’s recommendations. In brief, mRNA was purified from total RNA using poly-T oligo-attached magnetic beads. Fragmentation was conducted using divalent cations in NEBNext First Strand Synthesis Reaction Buffer (5 ×) under elevated temperature. First-strand cDNA was synthesized using random hexamer primer and RNase H, and second-strand cDNA synthesis was subsequently performed using dNTPs, DNA polymerase I, and RNase H. The library fragments were purified using QiaQuick PCR kits, and eluted with EB buffer. Then, terminal repair, A-tailing, and adapter were implemented. The libraries were sequenced on an Illumina platform and 150-bp paired-end reads were generated. Finally, about 6 Gb of clean data was obtained for each sample. The genome sequence of *P. ostreatus* PC15 strain was used as a reference genome (NCBI accession PRJNA81933, Riley et al. 2014). The obtained clean data were aligned to the reference genome using HISAT2 v2.1.0, the successor to TopHat2, which uses a modified BWT algorithm to convert reference genomes to index at a faster speed and with fewer resources. The FPKM value was used to define the expression level of each gene, and DESeq 2 was employed to determine the differentially expressed genes (DEGs) among treatments (adjusted *P *≤ 0.05 and |log2fold change | ≥ 1). The number of DEGs used to make a Venn diagram was ascertained, and the fold change values of all genes were subjected to KEGG enrichment analysis using the clusterProfiler R package (GSEA method) (Yu et al. 2012). The generated *P* values were adjusted using the BH method (Yu et al. 2012).

### Quantitative real-time PCR

Each treatment and each tissue comprised three biological replicates, and each biological replicate consisted of three mushrooms. The total RNA from each organ of the mushroom was extracted using TRIzol reagent (Invitrogen). The RNA was treated with DNaseI (Invitrogen), reverse-transcribed using SuperScriptTM RNase H-Reverse Transcriptase (Invitrogen), and then subjected to quantitative real-time PCR (qRT-PCR) using gene-specific primers (Additional file 1: Table S1 online). Amplification of the target gene during each cycle was monitored by using SYBR Green, and *Actin* (ID gene_9983) and *GPDH* (gene_10642) mRNA amplifications were used as internal quantitative control. The relative expression of the target genes was calculated using the △△Ct method (Livak and Schmittgen 2001).

### Statistical analysis

Differences in fresh weight and qRT-PCR results was determined by *t* test using SPSS version 16.0 (IBM). Statistical significance was set at *P *< 0.05. The statistical test for RNA-Seq data was performed using the DESeq 2 R package.

## Results

### Organ growth and number of DEGs

**Fig. 1:**
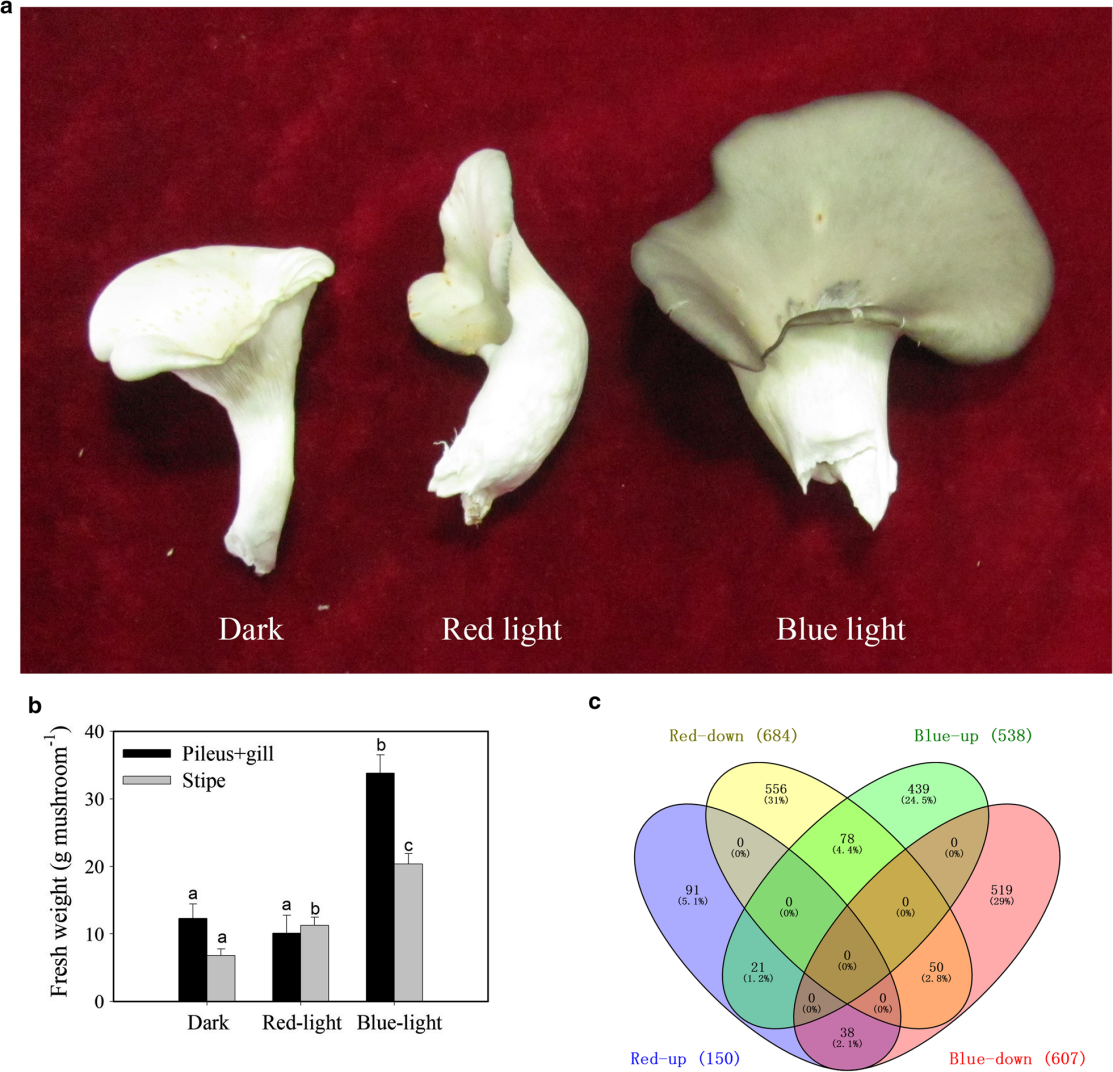
Effects of blue light and red light on growth (**a**, **b**) and gene expression (**c**) of *P. ostreatus*. Values of fresh weight are expressed as means (± standard deviation, SD) of 10 mushrooms. Means followed by different letters among treatments in the same organ are significantly different, according to *t* test (*P* < 0.05).Venn diagram showing the number of DEGs in the pileus between the red-light or blue-light treatment and dark treatment. DEGs were defined as genes with fold change > 2 and adjusted *P *≤ 0.05

Blue light increased the fresh weights of the stipe and pileus + gill of *P. ostreatus* (Fig. 1). Red light decreased pileus + gill fresh weight by 22%, although this reduction was not statistically significant (Fig. 1). In contrast, red light significantly enhanced the fresh weight of the stipe (Fig. 1). A Venn diagram showed that 538 and 150 genes were significantly upregulated in the pileus by blue light and red light, respectively, when compared with those by dark treatment. Among these upregulated genes, 21 genes were commonly upregulated following blue-light and red-light treatments. A total of 607 and 684 genes were significantly downregulated in the pileus by blue light and red light, respectively, among which 50 genes were commonly downregulated following the two treatments (Fig. 1c). In the stipe, 551 and 368 genes were significantly upregulated by blue light and red light, respectively, with 76 shared upregulated genes in both treatment groups. In contrast, 806 and 618 genes were significantly downregulated in the stipe by blue light and red light, respectively, with 324 shared downregulated genes in both treatment groups (Additional file 2: Figure S1). In the gill, only a very few DEGs were detected (Additional file 3: Figure S2).

### KEGG enrichment of DEGs

**Fig. 2:**
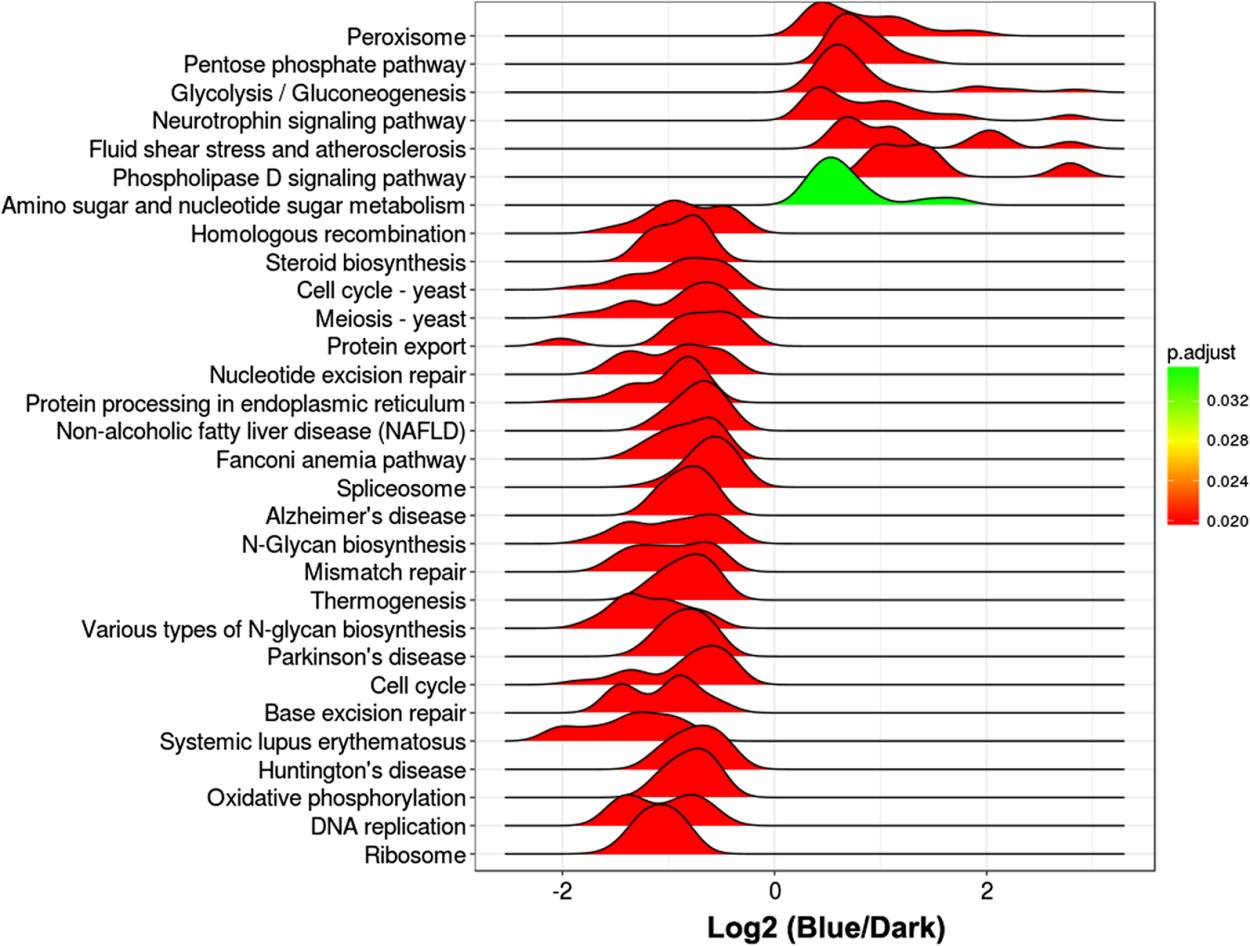
KEGG enrichment of different gene expressions in the pileus between blue-light treatment and dark treatment. Fold changes (blue-light/dark) of all the expressed genes were subjected to GSEA-KEGG enrichment

**Fig. 3:**
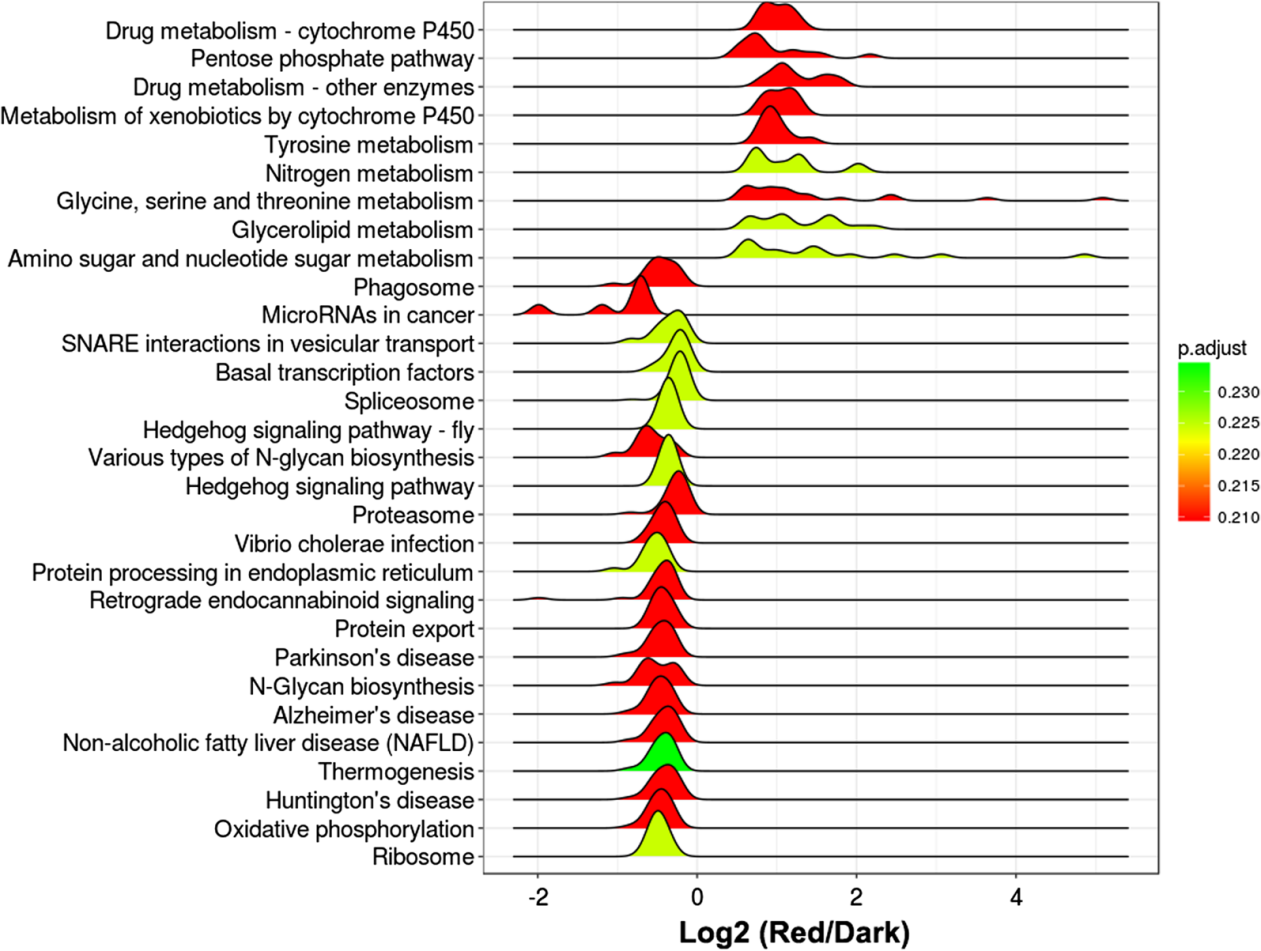
KEGG enrichment of different genes expressions in the pileus between red-light treatment and dark treatment. Fold changes (red-light/dark) of all the expressed genes were subjected to GSEA-KEGG enrichment

The fold change of all the expressed genes was subjected to GSEA-KEGG enrichment analysis, which reflects a comprehensive response of each pathway to different treatments. When compared with dark treatment, blue-light treatment significantly affected 31 pathways (adjusted *P* < 0.05) (Fig. 2) in the pileus, including 24 downregulated pathways and 7 upregulated pathways (Fig. 2). The upregulated pathways in the pileus following blue-light treatment included the peroxisome, pentose phosphate pathway, and glycolysis/gluconeogenesis (Fig. 2). Furthermore, analysis of differential gene expression in the pileus between blue-light and red-light treatments showed 40 pathways were significantly affected (adjusted *P* < 0.05) (Additional file 4: Figure S3), including 25 downregulated pathways and 15 upregulated pathways (Additional file 4: Figure S3). When compared with red-light treatment, the top five upregulated pathways in the pileus following blue-light treatment were the pentose phosphate pathway, glycolysis/gluconeogenesis, glycerolipid metabolism, peroxisome, and amino sugar and nucleotide sugar metabolism (Additional file 4: Figure S3). However, no statistically significant differences were noted among the pathways in the pileus between red-light and dark treatments (Fig. 3). In the stipe, although DEGs between blue-light and dark treatments as well as between blue-light and red-light treatments were enriched in many pathways, none of the enriched pathways were significantly affected (Additional file 5: Figure S4 and Additional file 6: Figure S5). In the gill, all enriched pathways of DEGs between blue-light and dark treatments were also not significantly affected (Additional file 7: Figure S6).

### Gene expression

**Fig. 4:**
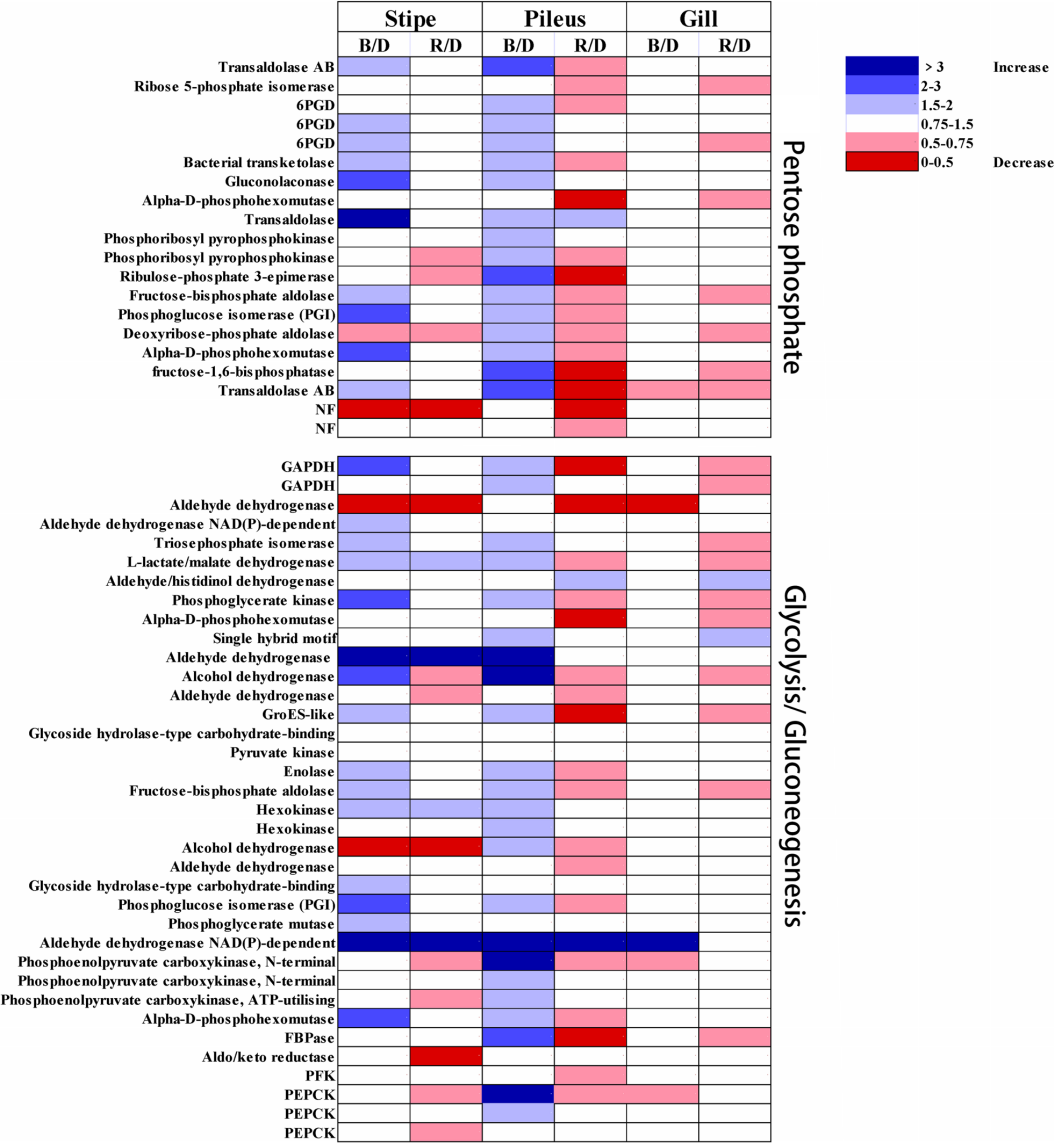
Fold changes of all the expressed genes involved in the pentose phosphate pathway and glycolysis/gluconeogenesis. FPKM values of all the genes involved in the pentose phosphate pathway and glycolysis/gluconeogenesis were used to calculate the fold changes between blue-light treatment/dark treatment (B/D) as well as red-light treatment/dark treatment (R/D). GAPDH, glyceraldehyde-3-phosphate dehydrogenase; PFK, 6-phosphofructokinase; 6PGD, 6-phosphogluconate dehydrogenase; PEPCK, phosphoenolpyruvate carboxykinase

**Fig. 5:**
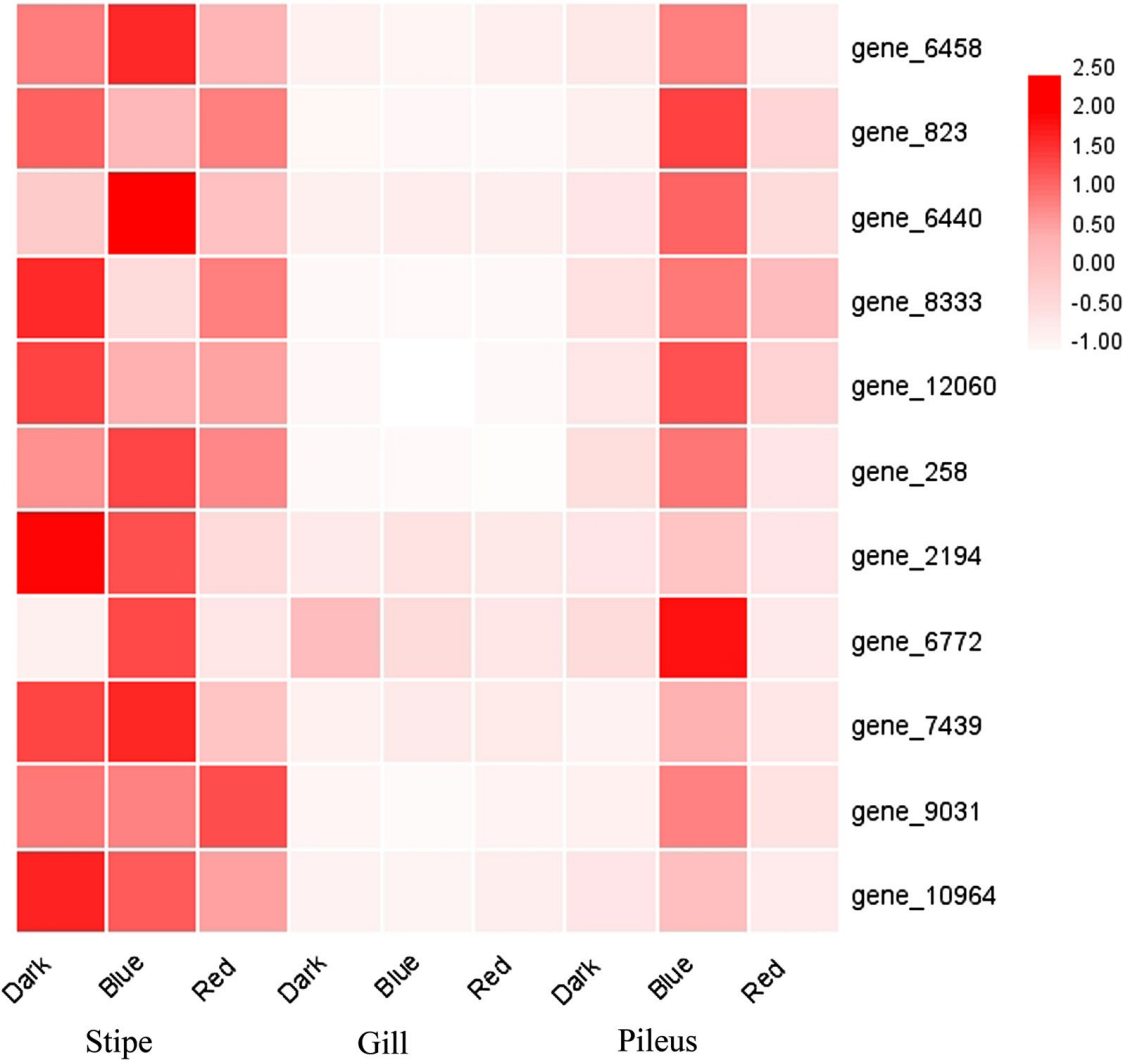
Heatmap showing expression of significantly upregulated serine/threonine protein kinase genes in the pileus following blue-light treatment. When compared with dark treatment, all the 11 serine/threonine protein kinase genes were significantly upregulated in the pileus by blue-light treatment (fold change > 2, adjusted *P *≤ 0.05)

In the present study, we particularly focused on serine/threonine protein kinase genes and the genes involved in the pentose phosphate pathway and glycolysis/gluconeogenesis. First, the FPKM values of all the genes involved in the pentose phosphate pathway and glycolysis/gluconeogenesis were determined, and the fold changes between blue-light treatment/dark treatment as well as red-light treatment/dark treatment were ascertained. Finally, these fold changes were employed to develop a heatmap to show the gene expression response to blue light and red light (Fig. 4 and Additional file 8: Table S2). As illustrated in Fig. 4, most of the genes involved in glycolysis/gluconeogenesis and the pentose phosphate pathway were upregulated in the pileus by blue light; some of these genes were also upregulated by blue light in the stipe (Fig. 4 and Additional file 8: Table S2). When compared with dark treatment, many genes involved in glycolysis/gluconeogenesis and the pentose phosphate pathway were downregulated in the pileus by red light, and a few genes were downregulated in the gill and stipe by red light (Fig. 4 and Additional file 8: Table S2). Many of the genes upregulated by blue light in the pileus were noted to be critical genes in glycolysis/gluconeogenesis and the pentose phosphate pathway, such as *6*-*phosphogluconate dehydrogenase (6PGD), glyceraldehyde*-*3*-*phosphate dehydrogenase (GAPDH)*, and *phosphoenolpyruvate carboxykinase (PEPCK)* (Fig. 4 and Table S2). In addition, blue light significantly upregulated 11 serine/threonine protein kinase genes (gene_6458, gene_823, gene_6440, gene_8333, gene_12060, gene_258, gene_2194, gene_6772, gene_7439, gene_9031, and gene_10964) in the pileus (Fig. 5). Figure 5 shows the FPKM values of all these significantly upregulated serine/threonine protein kinase genes (fold change > 2, adjusted *P *≤ 0.05) on a heatmap.

### qRT-PCR

The transcriptomics results were validated by qRT-PCR (Additional file 1: Table S1). The significant positive Pearson’s correlation coefficient between the transcriptomics and qRT-PCR results (R^2^ = 0.6688, *P* = 0.004) revealed that the transcriptomics fold-change values were similar to those of qRT-PCR data (Additional file 1: Table S1), thus indicating that the transcriptomics data were reliable.

## Discussion

We observed that blue light strongly stimulated the growth of all organs of *P. ostreatus,* especially pileus growth, while red light slightly (non-significantly) inhibited pileus growth (Fig. 1). Comparative transcriptomics analysis showed that the genes upregulated by blue light were significantly enriched in glycolysis/gluconeogenesis, the pentose phosphate pathway, and the peroxisome in the *P. ostreatus* pileus, but not in the gill or stipe, when compared with those observed following red-light and dark treatments (Fig. 1). Analysis of the fold change of all the expressed genes involved in glycolysis/gluconeogenesis and the pentose phosphate pathway revealed that most of these genes were upregulated in the pileus by blue light; some of these genes were also upregulated in the stipe by blue light (Fig. 4 and Table S2). Many of these upregulated genes are critical for glycolysis/gluconeogenesis and the pentose phosphate pathway. For example, *6PGD* is a rate-controlling gene of the pentose phosphate pathway, *GAPDH* is crucial in glycolysis, and *PEPCK* is the rate-controlling gene of gluconeogenesis (Méndez-Lucas et al. 2013, 2014). Similar studies have reported that blue light enhanced the accumulation of shikimic acid (Kojima et al. 2015) and the expression of numerous genes encoding CAZymes in mushrooms (Xie et al. 2018). In *P. ostreatus,* another important blue-light response is upregulation of genes involved in the peroxisome in the pileus. It must be noted that oxidases participating in this pathway are essential for many crucial metabolic processes such as fatty acid oxidation, biosynthesis of ether lipids, and free radical detoxification. Enhancement of the gene-encoding peroxisome could increase the metabolic activity of *P. ostreatus* under blue-light treatment. Besides, when compared with dark treatment, many genes involved in glycolysis/gluconeogenesis and the pentose phosphate pathway were downregulated in the pileus by red light, while only a few genes were downregulated in the gill and stipe by red light (Fig. 4). To date, no reports have shown that mushrooms perceive and respond to red light (Durand and Furuya 1985; Kitamoto and Gruen 1976; Sakamoto 2018); however, the results of the present study revealed that the expression of many genes in *P. ostreatus* was altered by red-light treatment, indicating that red light may be an important environmental factor that influences development of the fruiting body in mushrooms.

Taken together, blue light enhanced—whereas red light weakened—the activation of glycolysis and the pentose phosphate pathway. Glycolysis and the pentose phosphate pathway are two parallel respiration pathways in almost all organisms. Glycolysis is the basic and fundamental respiration pathway in all organisms, while the pentose phosphate pathway is an alternative glucose oxidizing pathway for NADPH generation. The pentose phosphate pathway is required for some reductive biosynthetic reactions such as cholesterol biosynthesis, bile acid synthesis, steroid hormone biosynthesis, and fatty acid synthesis (Kruger and Schaeweny 2003). The reducing equivalents and ATP utilized for biological metabolism in plants are generated through photosynthesis, glycolysis, and the pentose phosphate pathway. However, in mushrooms, photosynthesis is absent, and glycolysis and the pentose phosphate pathway drive the metabolic process by generating reducing equivalents and ATP. We believe that blue light improved *P. ostreatus* fruiting body (particularly, the pileus) growth through enhancement of glycolysis and the pentose phosphate pathway, whereas red light inhibited pileus growth by decreasing the activity of glycolysis and the pentose phosphate pathway.

Subsequently, we examined the molecular mechanisms underlying enhanced *P. ostreatus* biomass following blue-light treatment. Sensing blue light using blue-light photoreceptors is the first step in the blue-light response of mushrooms. To the best of our knowledge, the blue-light photoreceptor, phototropin-like protein White Collar-1 (WC-1), has been well described in the ascomycete *Neurospora crassa* (Sano et al. 2009). WC-1 is an essential component for all known blue-light responses of mushrooms, including biosynthesis of carotenoids in mycelia, formation of vegetative spores, and resetting of the circadian clock (Sano et al. 2009). Phototropins contain the serine/threonine protein kinase domain (Inoue et al. 2010; Lariguet and Dunand 2005). In the present study, 11 serine/threonine protein kinase genes were significantly upregulated in the *P. ostreatus* pileus by blue-light treatment (Fig. 5), some of which may be candidate blue-light photoreceptor genes, which should be further identified in future studies.

Transcriptomics is a powerful high-throughput approach to determine candidate genes for further investigation of underlying genetic mechanism and production of desired products. Based on the transcriptomics data obtained in this study, the following three suggestions are proposed for mushroom production or investigation of the mushroom blue-light response:

1. Blue light was found to produce positive effects on fruiting body development in *P. ostreatus,* but exert diverse effects on different organs; in contrast, red light had negative effects on fruiting body induction. Therefore, single-frequency light or mixed treatment comprising red light and blue light at different proportions could be used to produce more pilei and stipites.
2. To improve the production of mushrooms, more attention must be paid to glycolysis and the pentose phosphate pathway. Transgenic maneuvers of rate-controlling genes of glycolysis and the pentose phosphate pathway may yield exceptional improvements in mushroom production.
3. Although blue-light photoreceptor WC-1 has been well described in *N. crassa* (Sano et al. 2009), the vast species diversity among mushrooms may considerably amplify the complexity of the blue-light response. Different mushroom species may employ distinct molecular mechanisms to perceive and respond to blue light. The 11 serine/threonine protein kinase genes detected in the present study could be possible candidate genes encoding blue-light photoreceptors.

## Supplementary information

**Additional file 1: Table S1.** Results and primer sequences of qRT-PCR. PKG, pileus dark treatment; PBG, pileus blue-light treatment. Each treatment comprised three biological replicates.

**Additional file 2: Figure S1.** Venn diagram showing the number of DEGs in the stipe between the red-light or blue-light treatment and dark treatment. DEGs were defined as genes with fold change > 2 and adjusted *P *≤ 0.05

**Additional file 3: Figure S2.** Venn diagram showing the number of DEGs in the gill between the red-light or blue-light treatment and dark treatment. DEGs were defined as genes with fold change > 2 and adjusted *P *≤ 0.05

**Additional file 4: Figure S3.** KEGG enrichment of different genes expressions in the pileus between the blue-light treatment and red-light treatment. Fold changes (blue light/red light) of all the expressed genes were subjected to GSEA-KEGG enrichment.

**Additional file 5: Figure S4.** KEGG enrichment of different genet expressions in the stipe between the blue-light treatment and dark treatment. Fold changes (blue light/dark) of all the expressed genes were subjected to GSEA-KEGG enrichment.

**Additional file 6: Figure S5.** KEGG enrichment of different genet expressions in the stipe between the blue-light treatment and red-light treatment. Fold changes (blue light/red light) of all the expressed genes were subjected to GSEA-KEGG enrichment.

**Additional file 7: Figure S6.** KEGG enrichment of different gene expressions in the gill between the blue-light treatment and dark treatment. Fold changes (blue light/dark) of all the expressed genes were subjected to GSEA-KEGG enrichment.

**Additional file 8: Table S2.** Fold change values and *P* values of all the expressed genes involved in the pentose phosphate pathway and glycolysis/gluconeogenesis

## Data Availability

Oyster mushroom (*P. ostreatus*) strain zaoqiu615 is available from the corresponsing author upon request. All raw sequencing data have been deposited at NCBI (Accession Number PRJNA589667).

## Abbreviations

GAPDH: glyceraldehyde-3-phosphate dehydrogenase
PFK: 6-phosphofructokinase
6PGD: 6-phosphogluconate dehydrogenase
PEPCK: phosphoenolpyruvate carboxykinase
