# Supplementary figures and images for: Transcriptomic profiling sheds light on the blue-light and red-light response of oyster mushroom (Pleurotus ostreatus)
Source: AMB Express. 2020 Jan 18;10:10. doi: 10.1186/s13568-020-0951-x (PMC6969877; doi:10.1186/s13568-020-0951-x)

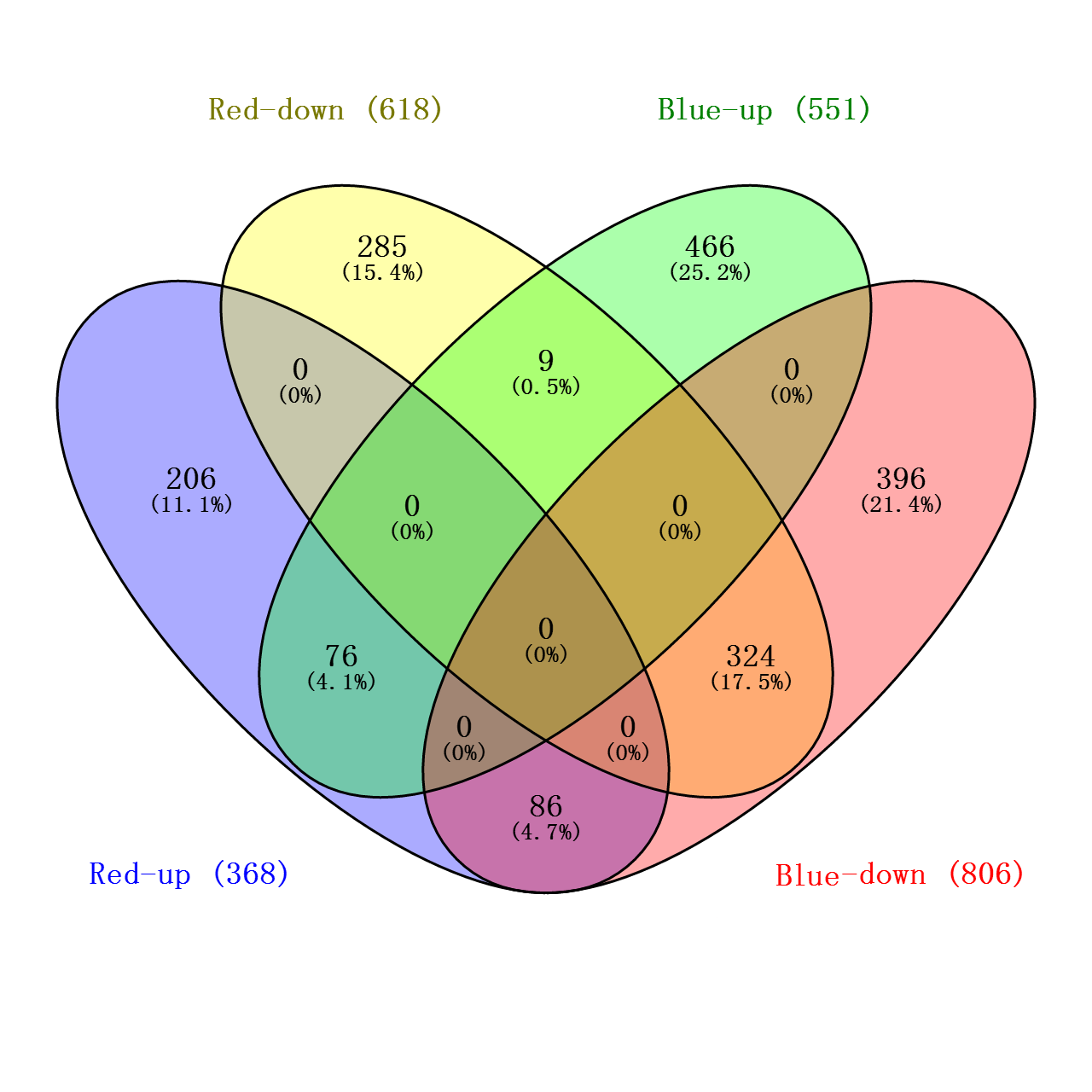

Supplement: Supplementary file 2 — Additional file 2: Figure S1. Venn diagram showing the number of DEGs in the stipe between the red-light or blue-light treatment and dark treatment. DEGs were defined as genes with fold change > 2 and adjusted P ≤ 0.05 [file 13568_2020_951_MOESM2_ESM.png]

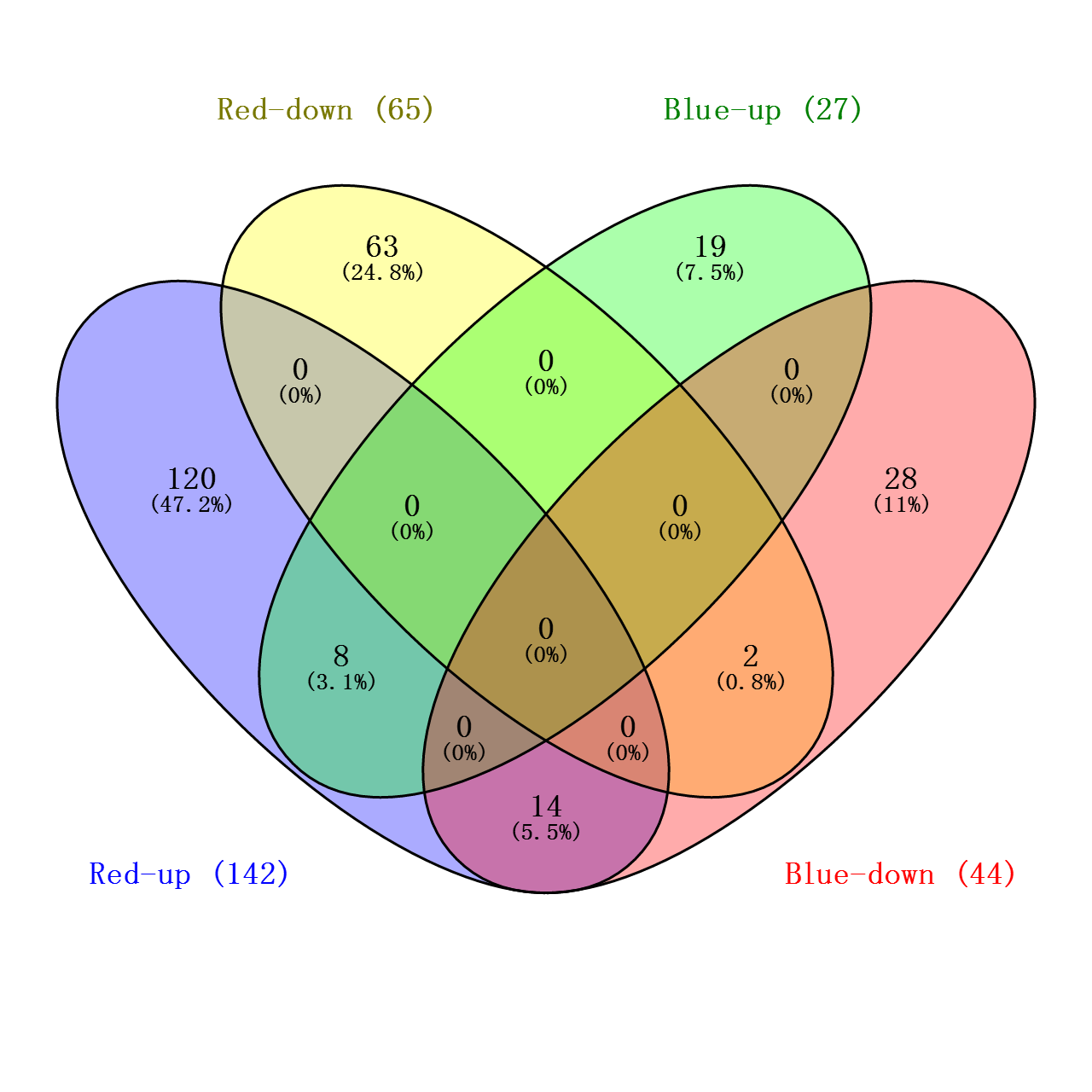

Supplement: Supplementary file 3 — Additional file 3: Figure S2. Venn diagram showing the number of DEGs in the gill between the red-light or blue-light treatment and dark treatment. DEGs were defined as genes with fold change > 2 and adjusted P ≤ 0.05 [file 13568_2020_951_MOESM3_ESM.png]

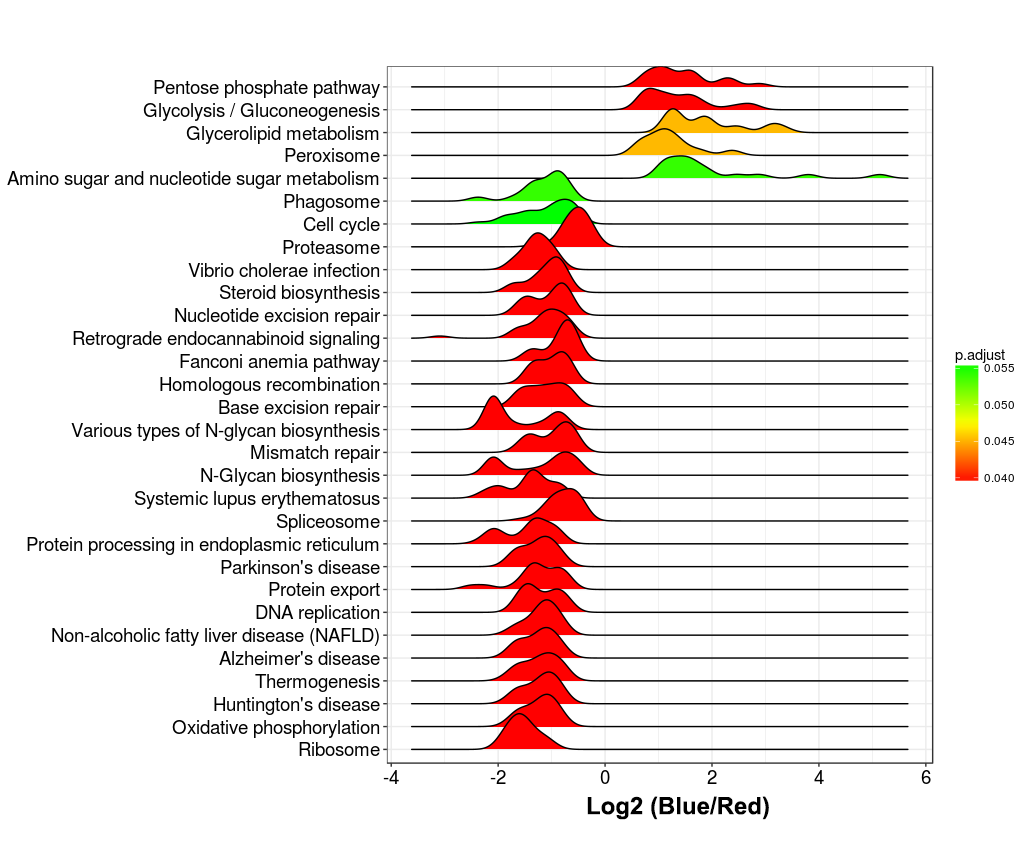

Supplement: Supplementary file 4 — Additional file 4: Figure S3. KEGG enrichment of different genes expressions in the pileus between the blue-light treatment and red-light treatment. Fold changes (blue light/red light) of all the expressed genes were subjected to GSEA-KEGG enrichment. [file 13568_2020_951_MOESM4_ESM.tif]

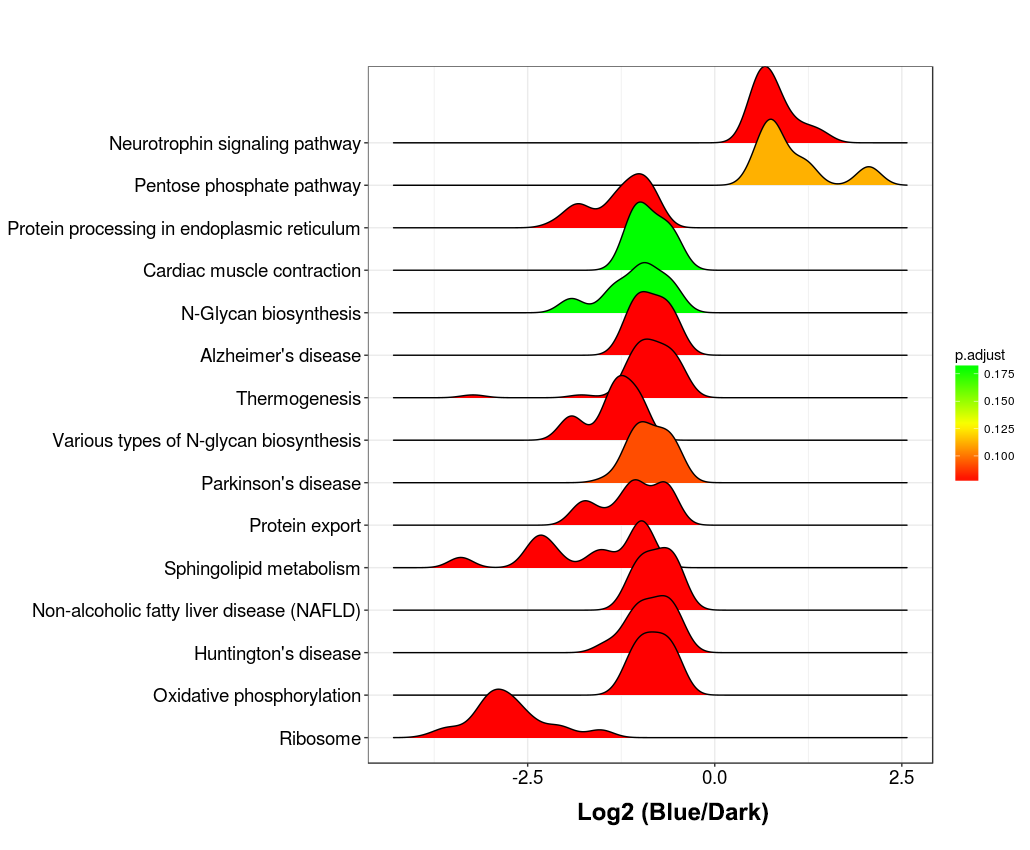

Supplement: Supplementary file 5 — Additional file 5: Figure S4. KEGG enrichment of different genet expressions in the stipe between the blue-light treatment and dark treatment. Fold changes (blue light/dark) of all the expressed genes were subjected to GSEA-KEGG enrichment. [file 13568_2020_951_MOESM5_ESM.tif]

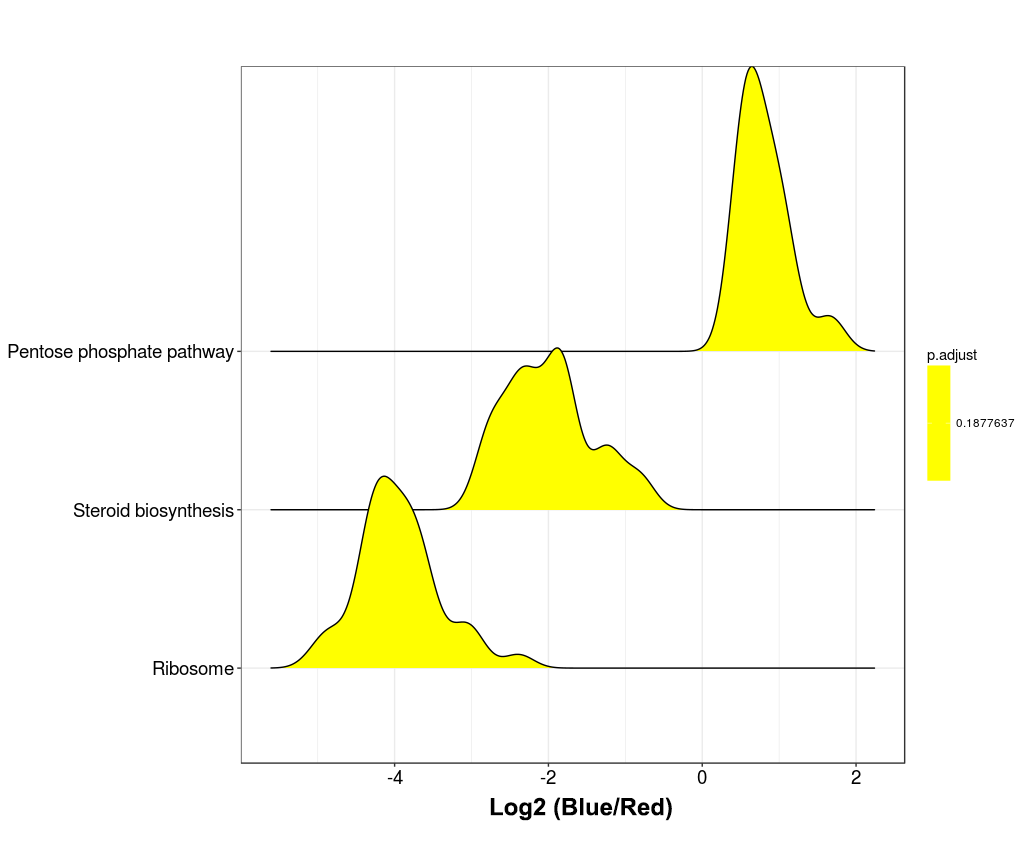

Supplement: Supplementary file 6 — Additional file 6: Figure S5. KEGG enrichment of different genet expressions in the stipe between the blue-light treatment and red-light treatment. Fold changes (blue light/red light) of all the expressed genes were subjected to GSEA-KEGG enrichment. [file 13568_2020_951_MOESM6_ESM.tif]

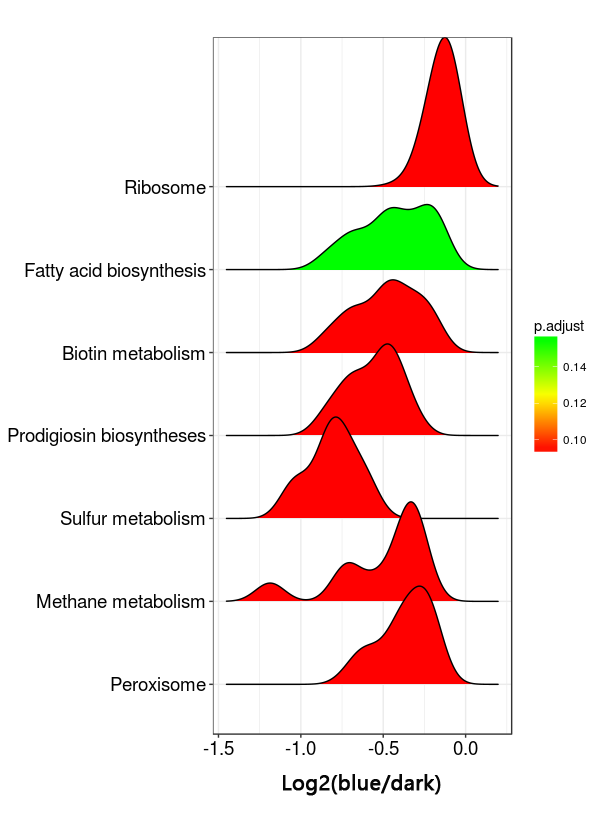

Supplement: Supplementary file 7 — Additional file 7: Figure S6. KEGG enrichment of different gene expressions in the gill between the blue-light treatment and dark treatment. Fold changes (blue light/dark) of all the expressed genes were subjected to GSEA-KEGG enrichment. [file 13568_2020_951_MOESM7_ESM.tif]
